# Addressing depression and comorbid health conditions through solution-focused brief therapy in an integrated care setting: a randomized clinical trial

**DOI:** 10.1186/s12875-024-02561-8

**Published:** 2024-08-23

**Authors:** Zach W Cooper, Orion Mowbray, Mohammed K. Ali, Leslie C. M. Johnson

**Affiliations:** 1 School of Social Work, University of Georgia, Williams Street, Atlanta, GA 30602 Georgia; 2 Department of Family and Preventative Medicine, School of Medicine, Emory University, Atlanta, Georgia; 3 Emory Global Diabetes Research Center of the Woodruff Health Sciences Center, Emory University, Atlanta, Georgia

**Keywords:** Behavioral intervention, Depression, Primary care, Integrated behavioral health, Comorbid disorders

## Abstract

**Background:**

Co-occurring physical and mental health conditions are common, but effective and sustainable interventions are needed for primary care settings.

**Purpose:**

Our paper analyzes the effectiveness of a Solution-Focused Brief Therapy (SFBT) intervention for treating depression and co-occurring health conditions in primary care. We hypothesized that individuals receiving the SFBT intervention would have statistically significant reductions in depressive and anxiety symptoms, systolic blood pressure (SBP), hemoglobin A1C (HbA1c), and body mass index (BMI) when compared to those in the control group. Additionally, we hypothesized that the SFBT group would have increased well-being scores compared to the control group.

**Methods:**

A randomized clinical trial was conducted at a rural federally qualified health center. Eligible participants scored ≥ 10 on the Patient Health Questionnaire (PHQ-9) and met criteria for co-occurring health conditions (hypertension, obesity, diabetes) evidenced by chart review. SFBT participants (*n* = 40) received three SFBT interventions over three weeks in addition to treatment as usual (TAU). The control group (*n* = 40) received TAU over three weeks. Measures included depression (PHQ-9) and anxiety (GAD-7), well-being (Human Flourishing Index), and SFBT scores, along with physical health outcomes (blood pressure, body mass index, and hemoglobin A1c).

**Results:**

Of 80 consented participants, 69 completed all measures and were included in the final analysis. 80% identified as female and the mean age was 38.1 years (SD = 14.5). Most participants were white (72%) followed by Hispanic (15%) and Black (13%). When compared to TAU, SFBT intervention participants had significantly greater reductions in depression (baseline: M = 18.17, SD = 3.97, outcome: M = 9.71, SD = 3.71) and anxiety (baseline: M = 14.69, SD = 4.9, outcome: M = 8.43, SD = 3.79). SFBT intervention participants also had significantly increased well-being scores (baseline: M = 58.37, SD = 16.36, outcome: M = 73.43, SD = 14.70) when compared to TAU. Changes in BMI and blood pressure were not statistically significant.

**Conclusion:**

The SFBT intervention demonstrated efficacy in reducing depressive and anxiety symptoms and increasing well-being but did not affect cardio-metabolic parameters over a short period of intervention.

**Trial Registration:**

The study was pre-registered at ClinicalTrials.gov Identifier: NCT05838222 on 4/20/2023. *M = Mean, SD = Standard deviation.

**Supplementary Information:**

The online version contains supplementary material available at 10.1186/s12875-024-02561-8.

## Background

Depressive disorders are prevalent and have deleterious effects on health including functional impairment and increased mortality [1, 2]. Depression frequently accompanies chronic illnesses including diabetes [3, 4], and hypertension [5–7]. For example, a recent study finds that 69% of patients with diabetes have anxiety, 65% have depression, 62% reported a fear of low blood glucose, and half reported no discussion of mental health (MH) with their medical care team [8]. Co-occurring medical and MH disorders have a bidirectional influence on physical and mental health (MH) outcomes meaning that MH symptoms can create or exacerbate physical health problems and physical health symptoms can lead to or exacerbate MH symptoms [4, 5, 9, 10]. Studies have shown negative health outcomes are mediated by negative health behaviors including nonadherence to treatment recommendations (e.g. diet, exercise, medication compliance) [11, 12]. Consequently, addressing MH and medical conditions simultaneously is essential for quality primary healthcare [3, 13].

Despite the prevalence of co-occurring disorders and the connection between MH and health outcomes, healthcare remains fragmented [14]. Only 10% of patients with a MH disorder receive treatment from a MH specialist and 57% receive no treatment [15]. The remaining 33% are treated by primary care providers (PCPs), but many PCPs cite a lack of time and expertise as barriers to treating MH conditions [16–18]. Integrated care (IC) models have emerged to increase access to behavioral health care [14, 19, 20]. IC models support PCPs in treating MH through a team approach incorporating a behavioral health clinician (BHC) (e.g., clinical social worker, psychologist) within the traditional primary care team [14, 19]. To align their workflow with the primary care system (15–30 min sessions; population health emphasis), BHCs informally adapt evidence-based interventions [14, 21]. Within IC models such as the primary care behavioral health (PCBH) model, interventions can occur within a single session, and are most frequently conducted within treatment episodes of 1–3 visits [21]. Currently, there are few Randomized Clinical Trials (RCTs) that have operationalized and tested behavioral interventions for IC models. Our study addresses this gap by adapting a Solution Focused Brief Therapy (SFBT) intervention for an IC model. Specifically, the intervention was designed to be utilized within a PCBH model (20–30 min in length, 1–3 sessions).

SFBT interventions are flexible, efficient, and evidenced-based, and therefore fit the primary care workflow. The unique foundations of SFBT interventions include (1) focusing on strengths, (2) collaborating with patients to identify their preferred future, and (3) identifying and mobilizing patient strengths to move toward that preferred future [22]. SFBT interventions have been tested within medical settings for addressing obesity and promoting health behaviors within primary care settings, and SFBT interventions have demonstrated efficacy for addressing depressive symptoms [23, 24]. However, there are no RCTs evaluating SFBT interventions for IC models within primary care settings to address depression with co-occurring chronic illnesses. Our study addresses this gap. The objective of this study was to examine the efficacy of SFBT intervention for addressing depression, and co-occurring health disorders (primary outcomes), and for addressing anxiety and improving overall well-being (secondary outcomes). We hypothesize that individuals in the SFBT group will have significant reductions in depressive and anxiety symptoms, systolic blood pressure (SBP), and body mass index (BMI) when compared to the control group. We also hypothesize that individuals receiving the SFBT intervention will have increased well-being scores when compared to the control group.

## Methods

### Study design

This study employed a single-site RCT design utilizing block randomization in groups of 20 at the patient level. The trial took place at a large, rural Federal Qualified Health Center (FQHC) located in the Southeastern region of the United States. All procedures adhered to federal guidelines for the ethical treatment and safeguarding of human subjects and received approval from the Institutional Review Board (IRB) in the Southeastern United States.

### Study population

Patients were eligible for inclusion in the study if they were: (1) 18 years or older, (2) proficient in English, (3) scored ≥ 10 on the Patient Health Questionnaire (PHQ-9), and (4) had at least one co-occurring health condition (i.e., hypertension, obesity, diabetes). Exclusion criteria were deliberately minimized to enhance applicability and pragmatism and encompassed: (1) current suicidal ideation, (2) prior participation in solution-focused (SF) treatment, and (3) inability to comprehend the informed consent process.

### Recruitment

Patients were recruited via a registry report produced by clinic staff including all patients who had scored ≥ 10 on the PHQ-9 within the past 5 months. The report yielded 373 unique patients. After eliminating patients who did not meet the inclusion criteria (e.g., under 18, non-English speaking, no co-occurring medical diagnosis), there were a total of 142 eligible participants. These eligible participants were contacted via phone calls and secure messaging via the patient portal to acquire the 80 participants included.

### Study randomization and treatment

#### Randomization

**Fig. 1:**
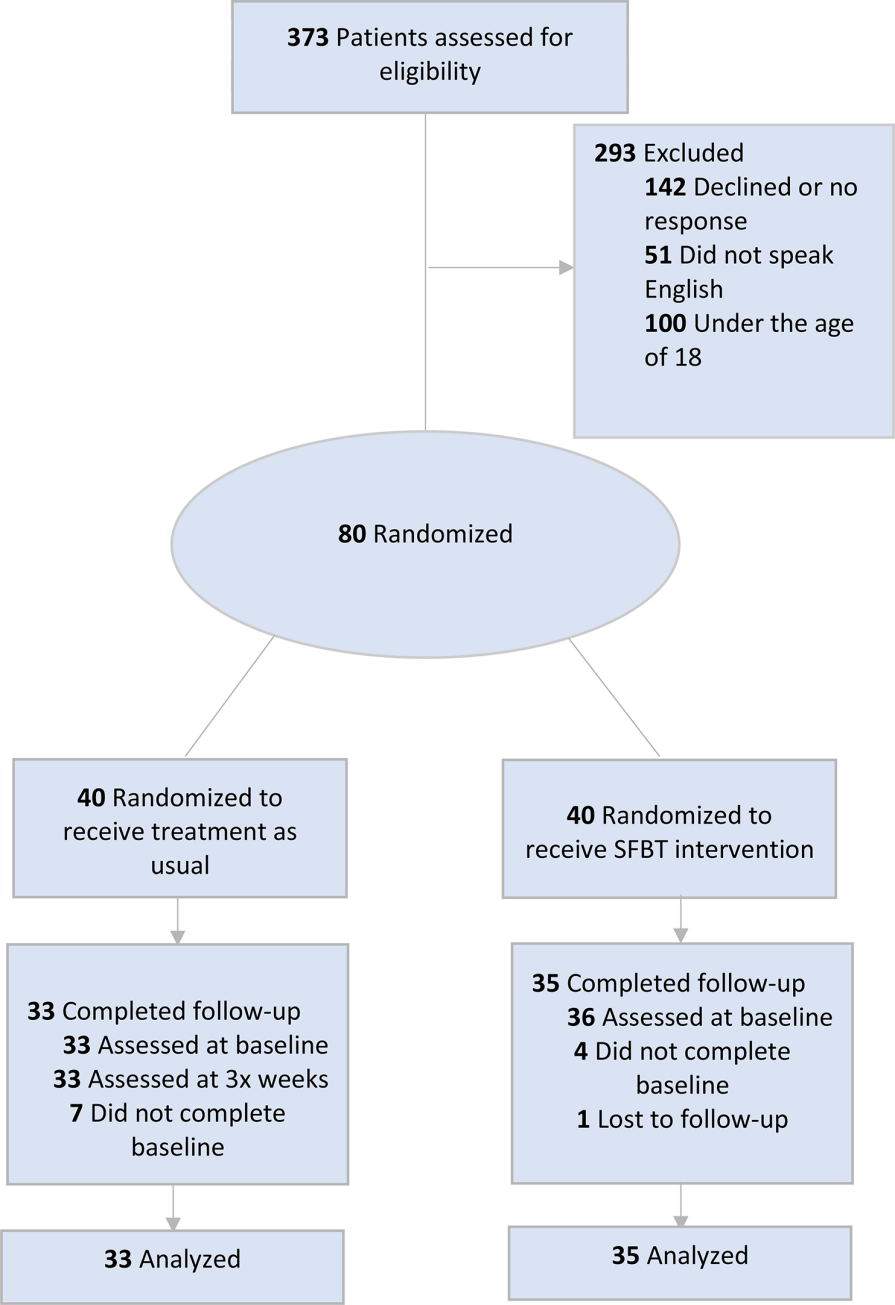
CONSORT diagram

Upon obtaining informed consent, participants were enrolled as active participants. The process of block randomization was employed, involving groups of 20 participants each. As soon as 20 participants were recruited, they were randomized into either the treatment or control group. This approach led to a final count of 40 participants in the control group and 40 participants in the treatment group. The large block size of 20 participants was employed to increase randomness and acquire balance between the treatment and control groups. Participants and the researcher who mentored statistical analyses were blinded to treatment condition. For a full description of the recruitment and randomization process, please refer to Fig. 1 for the full CONSORT diagram.

#### Intervention components

Participants in both the SFBT intervention and treatment as usual (TAU) groups had standardized interactions with the interventionist. Both groups (1) had the same frequency and duration of contact with the interventionist, (2) were informed that they would either experience a SFBT intervention or an alternative intervention, (3) were provided with all the same screening measures, (4) were educated regarding the importance of random assignment and maintaining blindness throughout the study, and (5) were provided access to the same informed consent and descriptions of the study protocol.

TAU included visits with PCPs and medication for health conditions. Instead of receiving the SFBT intervention, participants in the control group engaged in a traditional, problem-focused psychological assessment; these participants were not provided with any behavioral intervention. Participants in the TAU group were educated on the importance of acquiring a history of present illness (HPI) before engaging in an intervention. Emphasizing the importance of assessment enabled the researchers to maintain blinding to assignment for those in the control group. The follow-up visit for those in the TAU group included further assessment of problems associated with their symptoms. The following week participants in the control group engaged in outcome screenings.

### Solution focused brief therapy intervention

The SFBT Intervention was adapted from a Solution-Focused Brief Therapy manual [22]. The primary author (also a licensed clinical social worker) assumed the role of the interventionist, leveraging their proficiency in the protocol and SFBT intervention. To address any potential allegiance bias, the interventionist used a random number generator procedure in Excel to facilitate randomization, shared the randomization procedures with another researcher to ensure accuracy, and had biweekly discussions with a co-author and researcher regarding ethical decision-making for the study. Participants were educated on the importance of maintaining blinding to intervention assignment. To maintain blinding for those in the intervention group, the interventionist performed the SFBT intervention without providing education or information regarding which intervention the participants were experiencing.

Those within the SFBT intervention arm attended a 20–30-minute individual visit following the baseline screening questions with the interventionist. Most visits were completed separately from the patient’s regular PCP visits. Each visit included the main active ingredients of SFBT treatment including: (1) the establishment of a collaborative, therapeutic environment, (2) the emphasis of a solution-focused approach, (3) establishing and setting measurable and attainable treatment goals, (4) utilizing future-oriented questions, (5) the utilization of scaling questions to analyze progress, and (6) the use of questions to elicit examples from the patient of when their problem was not present [22]. A charting template was utilized to maintain the same structure across initial interventions consisting of: (1) patient engagement and collaboration, (2) review of patient goals and values, (3) assessment of informal supports, 3) self-assessment of participants’ strengths, (4) utilization of change questions to identify change goals, and (5) operationalizing goals to be measurable and achievable. Initial sessions included 10–15 min for baseline screening. Future SFBT intervention sessions built on previous change goals, analyzed what was improved over the past week, and then identified methods to mobilize behavior change progress. Final visits included 20–30 min of an SFBT intervention followed by 10–15 min to perform the outcome screenings. Session length was tracked for each participant visit.

#### Intervention fidelity

Fidelity was comprehensively assessed by monitoring SFBT integrity, assessing the receipt of the SFBT intervention, and measuring the enactment of the SFBT intervention while maintaining the intervention structure and ensuring provider competence [25] For example, the SFBT fidelity monitoring tool was used to monitor intervention integrity throughout the study [26]. This self-report screening tool analyzes the degree to which the interventionist adhered to the structure of the SFBT intervention. The interventionist performed the screening after each visit with patients in the treatment group. In addition, the authors created an SFBT scale to analyze the enactment of the SFBT intervention via constructs such as hope, connection to important people, and awareness of strengths. A more detailed description of the fidelity strategies used in this study is reported separately [25].

#### Data collection and follow-up

Measures were collected on two occasions: at baseline and again after 3 weeks. For those in the intervention group, the initial SFBT intervention visit was conducted after the baseline screenings were completed to prevent the effect of the intervention from influencing baseline results. The outcome assessments were performed after the third SFBT intervention. The 3-week duration was chosen to mimic a brief treatment episode which is common within primary care settings that utilize IC models [14, 21]. See Supplement 1 for a graphical depiction of the data collection process.

#### Measures

Primary outcomes within the study were (1) symptoms of depression and (2) health outcomes. Secondary outcomes included (1) symptoms of anxiety, (2) well-being, and (3) SFBT attributes. Each is defined in greater detail below. The independent variable within the study was the receipt of the SFBT intervention compared with TAU.

##### Symptoms of depression

Depression was measured at both pre-test and post-test with the nine-Patient Health Questionnaire (PHQ-9) [27]. The PHQ-9 is a standard assessment utilized in all FQHCs and many primary care clinics. The PHQ-9 has an established history of predictive validity and acceptable sensitivity (88%) and specificity (88%) [27]. PHQ-9 scores were compared between the treatment and control groups to assess differences with depression.

##### Health outcomes

Health outcomes were assessed through patient chart review at both pre-test and post-test. These measures included traditional healthcare markers such as levels of blood pressure (both diastolic and systolic blood pressure), body mass index (BMI), and HbA1c measurements [28]. Differences were compared between the treatment and control groups to assess differences in health outcomes.

##### Symptoms of anxiety

Anxiety was measured at both pre-test and post-test with the general anxiety disorder-7 (GAD-7) scale [29]. The GAD-7 is a standard screening tool utilized in many FQHCs and primary care clinics. The GAD-7 has an established history of predictive validity and acceptable sensitivity (83%) and specificity (84%). GAD-7 scores were compared between the treatment and control groups to assess differences in anxiety.

##### Human flourishing

Flourishing was measured at pre-test and post-test with the Flourishing index [20]. The Flourishing index assesses several domains of flourishing including (1) life satisfaction, (2) mental and physical health, (3) meaning and purpose, (4) character and virtue, (5) close social relationships, and (6) financial and material stability [31]. The Flourishing index has demonstrated sufficient reliability (α = 0.89) and predictive validity [32]. These scores were compared between the treatment and control groups to assess differences in human flourishing.

##### Solution-focused attributes

The SFBT scale was provided at both pretest and posttest utilizing scaling questions assessing core SFBT constructs such as hope, self-perception of strengths, connection to important people, confidence to solve problems, and confidence that their future will be good. The reliability of the SFBT questions was good with a Cronbach alpha of 0.82 at baseline and an alpha of 0.82 for outcome measures. The scale had good face and content validity and was reviewed by SFBT experts. Predictive and criterion validity was tested by assessing whether individuals in the SFBI group had significant gains on the scale when compared to the control group, these statistics are reported in the results section.

##### Acceptability, feasibility, and appropriateness of intervention measure

The Acceptability, Feasibility and Appropriateness measures were created by Weiner and colleagues based on Proctor’s implementation outcomes [33, 34]. Each of the three scales consists of four items, utilizing a 5-point Likert scale ranging from “1” = completely disagree to “5” = completely agree to rate statements assessing each outcome. This scale has demonstrated good reliability (Cronbach alpha range 0.85 to 0.91) and validity for numerous studies using implementation science measures[33].

##### Co-occurring treatment

Researchers documented the use of medication and psychotherapy among patients in the control and treatment groups. Participants’ medical charts were reviewed at baseline, 2 weeks, and 3 weeks to assess whether individuals had received psychiatric medications, therapy, or both. Participants received a “1” for engaging in TAU, a “2” for engaging in pharmacotherapy and TAU, and a “3” for engaging in TAU, pharmacotherapy, and psychotherapy. This variable was included in our analysis as a control variable.

##### Demographic measures

These measures were collected only at baseline and included age as a continuous variable, gender as a dichotomous variable, race as a categorical variable, and income as a continuous variable.

### Statistical analysis

All statistical analyses were performed using intention-to-treat principles. We utilized a random number generator to facilitate randomization and withheld performing any analyses until all data was collected. Prior to data collection, a power analysis was conducted utilizing G*Power to determine the necessary sample size to perform a repeated measures factorial ANOVA. A sample of 48 participants was recommended to detect medium to small effects for depression, anxiety, and other health outcomes (d = 0.20). To account for potential loss to follow-up and to increase precision for detecting trending effects and smaller effect sizes, we sought to acquire a total of 80 patients, 40 in the treatment arm and 40 in the control arm. 69 patients were included in the intention-to-treat analysis, and 68 participants completed all study measures. Consequently, there is high confidence that all statistical analyses are sufficiently powered as 68 participants is well above the 48 participants needed to detect small to medium treatment effects.

T-tests examined any significant differences between the treatment and control groups at baseline and showed no differences in age, sex, or baseline health and MH scores. SFBT attributes was the only measurement construct that significantly varied at baseline. Though statistically significant, the clinical significance of this difference was minimal. In addition, sensitivity analyses were performed to ensure this baseline difference did not significantly influence the results.

A repeated-measures ANOVA was run to determine whether there was a significant difference in baseline and outcome scores between the treatment and control group. We utilized Bonferroni corrections when interpreting results to account for the multiple comparisons and post hoc analyses within our repeated-measures ANOVAs [35]. We also pre-registered our analytic approach and study hypotheses at ClinicalTrials.gov (Identifier: NCT05838222). Analyses were run utilizing SAS^®^. For each analysis, F statistics were provided to depict significance, effect sizes were provided via 95% confidence intervals (CIs) of mean differences between groups using a 2-sided statistical test and a significance threshold of *p* < .05.

In addition to mean differences, an eta-squared effect size (*n*^2^) is included in Table 1. This statistic explains how much of the change for each variable is explained by the SFBT intervention. For example, depression had an effect size of *n*^2^ = 0.25 meaning that 25% in the change of depression scores between the control and intervention group is explained by the SFBT intervention. See Supplement 2 for the trial protocol and prespecified analysis plan. The CONSORT guidelines were utilized to structure all reporting of reported results, see Supplement 3 for the CONSORT checklist.

**Table 1:** Baseline summary of demographic statistics

	No. (%)		
**Characteristic**	**Solution-Focused group** **(n = 36)**	**Treatment as usual group** **(n = 33)**	**Total** **(n = 69)**
**Sociodemographic characteristics**			
Age, mean (SD)	35.86 (13.92)	40.55 (14.96)	
**Sex**			
Men	6 (17%)	8 (24%)	14 (20%)
Women	30 (83%)	25 (76%)	55 (80%)
**Race/Ethnicity**			
White	26 (72%)	24 (72%)	50 (72%)
Black	2 (6%)	7 (21%)	9 (13%)
Hispanic	8 (22%)	2 (6%)	10 (15%)
**Household income, Based in poverty line**			
100% or below poverty line	15 (45%)	23 (64%)	38 (55%)
101-150% above poverty line	8 (24%)	6 (17%)	14 (20%)
151-199% above the poverty line	4 (12%)	2 (6%)	6 (9%)
200% or above the poverty line	1 (3%)	2 (6%)	3 (4%)
Missing	8 (24%)	0 (0%)	8 (12%)
**Clinical Characteristic**			
Depression, anxiety, well-being, and cardiovascular indices, mean (SD)			
PHQ-9 score	18.14 (3.92)	17.81 (4.60)	17.99 (4.23)
GAD-7 score	14.84 (4.40)	15 (3.93)	14.84 (4.40)
Flourishing score	58.61 (16.19)	53.48 (23.42)	56.24 (19.88)
Solution focused score	27.19 (9.78)	21.47 (11.46)	24.50 (10.91)
Body mass index (BMI)	34.66 (8.61)	33.56 (10.51)	34.1 (9.52)
**BP, mean (SD), mm Hg**			
Systolic	121 (15.15)	127.06 (16.83)	123.90 (15.15)
Diastolic	78.08 (8.95)	78.00 (8.94)	78.04 (8.95)

## Results

### Baseline

Among 80 participants who originally consented, 10 participants did not complete the initial baseline assessment. One additional participant from the treatment group was lost to follow-up after the second SFBT session. Of the 69 patients who successfully completed all assessment measures, 36 were in the treatment group and 33 were in the control group. The majority (79.7%) of the participants identified as female and the average age was 38 years old (*SD* = 14.5, range = 18–74). Most participants identified as white (72.5%) followed by Hispanic (149%), and then Black (13.1%). On average, patients reported moderately severe depressive symptoms as indicated by the PHQ-9 (M = 17.9, SD = 4.2). Regarding anxiety, participants were moderately anxious as indicated by the GAD-7 (M = 14.8, SD = 4.4). Participants averaged 56.2/100 (SD = 19.9) on the Flourishing index with higher scores indicating higher levels of flourishing. Regarding SFBT constructs, participants averaged 24.5/50 (SD = 10.9). For biological measures, patients had an average BMI of 34.1 (SD = 15.2) with an average blood pressure of 123.9 (SD = 15.2)/78.0 (SD = 8.9). See Table 2 for a full description of baseline demographic data.

**Table 2:** Clinical significance of depression outcomes

	Baseline		Post treatment	
PHQ-9 Severity Status	Control (*n* = 33)	Treatment (*n* = 36)	Control (*n* = 33)	Treatment (*n* = 35)
None (0–9)			1/33 (3%)	14/35 (40%)
Mild (10-14)	8/32 (25%)	10/36 (28%)	5/33 (15%)	18/35 (51%)
Moderate (15-19)	11/32 (34%)	13/36 (36%)	13/33 (40%)	3/35 (9%)
Severe (20+)	14/32 (41%)	13/36 (36%)	14/33 (42%)	0/35 (0%)
Missing Data	0/32 (0%)	0/36 (0%)	0/33 0%)	1/36 (3%)
Meet Criteria for MDD	32/32 (100%)	36/36 (100%)	32/33 (97%)	22/36 (61%)

### ANOVA results

For the 4 MH outcomes, compared with TAU, those in the SFBT intervention group had greater reductions in the primary outcome of depression scores (*F* [1, 65] = 20.7, *p* < .001): mean difference, -9.3 [95% CI, -7.2 to -11.3], see Table 3 for a summary of depression outcomes. Similarly, those in the SFBT intervention group had significantly larger reductions for secondary outcomes including anxiety scores when compared to TAU (*F* [1, 65] = 17.2, *p* < .001): mean difference, -7.6 [95% CI, -5.7 to -9.4]. Further, those in the SFBT intervention group had significantly greater improvement in scores on the Flourishing Index (secondary outcome) when compared to TAU (*F* [1, 65] = 10.1, *p* < .002): mean difference, 24.9 [95% CI, 15.7 to 33.9]. Last, the SFBT scale (secondary outcome) was analyzed. Those in the SFBT intervention group had significantly larger increases on the SFBT scale when compared to TAU (*F* [1, 64] = 22.7, *p* < .001): mean difference, 15.1 [95% CI, 11.2 to 18.9].

**Table 3:** Primary and secondary outcomes in a pilot RCT assessing effects of SFBT intervention

Outcome	Treatment as Usual	SFBT Group	Adjusted between-group difference (95% CI)	Sum of Squares	DF	F Value		Effect Size (*n*^2^)
**Depression**	*n* = 33	*n* = 35						
Baseline	17.76 (4.54)	18.17 (3.97)	0.41 (-2.48-1.65)	659.35	(1,66)	21.65**		0.25
Outcome	18.94 (4.75)	9.71 (3.72)	-9.41 (-7.17- -11.29)					
**Anxiety**	*n* = 33	*n* = 35						
Baseline	15.15	14.69	− 0.47 (-1.70-2.63)	548.60	(1,66)	18.36**		0.22
Outcome	15.97	8.43	-7.57** (-5.71- -9.43)					
**Well-Being**	*n* = 32	*n* = 35						
Baseline	53.48	59.37	5.50 (-4.26- 15.25)	7705.28	(1,66)	10.99*		0.15
Outcome	48.00	73.43	24.87** (15.74-34.0)					
**SFBT Constructs**	*n* = 32	*n* = 35						
Baseline	22.00	27.03	5.47* (-0.29-10.64)	3512.54	(1,66)	24.12**		0.27
Outcome	19.84	34.97	15.03** (11.21–18.86)					
**BMI**	*n* = 22	*n* = 26						
Baseline	34.33	35.43	1.11 (-4.70- 6.91)	12.61	(1,46)	0.063		0.001
Outcome	34.74	35.09	0.35 (-5.52-6.21)					
**Systolic Blood Pressure**	*n* = 23	*n* = 26						
Baseline	124	118.08	-5.92 (-13.53-1.67)	1033.37	(1,47)	3.70		0.07
Outcome	124.78	117.69	-7.09 (-15.85-1.67)					
**Diastolic Blood Pressure**	*n* = 23	*n* = 26						
Baseline	76.22	77.77	1.55 (-3.67- -6.77)	2.50	(1,47)	0.22		0.001
Outcomes	78.00	75.81	-2.19 (-7.83-3.44)					
**Hemoglobin A1C**	*n* = 3	*n* = 2						
Baseline	8.87	12.60	3.73* (-6.74- − 0.73)	19.15	[1, 3]	11.44		0.79
Outcomes	9.03	10.95	1.92 (-0.58-4.41)					

*p < .05

**p < .01

**Fig. 2:**
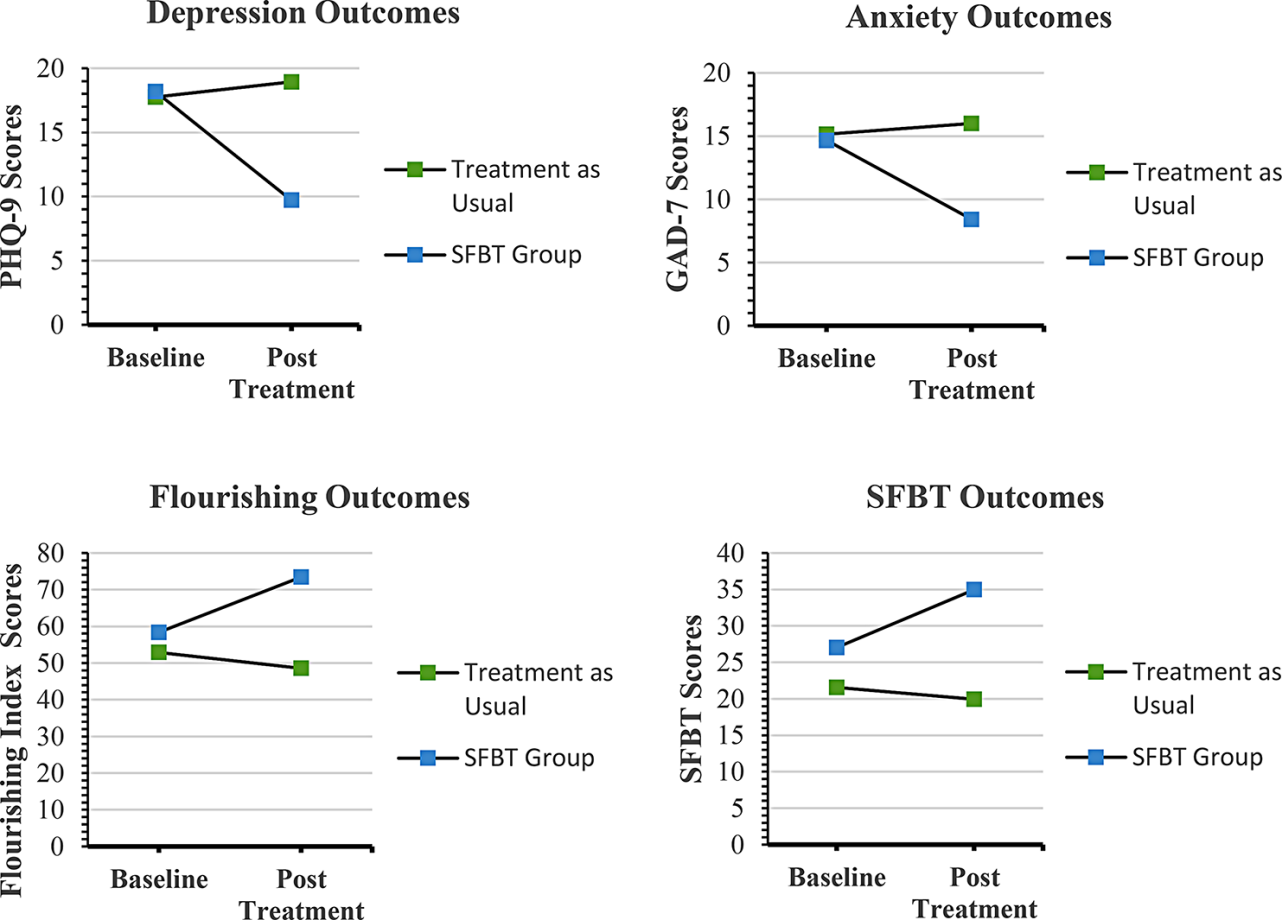
Summary of significant primary and secondary outcomes

Regarding the 4 primary medical outcomes, compared with TAU, there was a significant effect for HbA1c and trending effects for SBP. Those in the SFBT intervention group had greater reductions in HbA1c: mean difference, -1.9 [95% CI, − 0.58 to − 4.4]. Similarly, there were trending effects for SBP with those in the SFBI group having greater reductions in SBP when compared to TAU (*F* [1, 47] = 3.7, *p* < .060): mean difference, -7.1 [95% CI, 1.6 to -15.8]. Regarding changes in BMI, there was a significant within-group difference for those in the SFBI group (*F* [1, 46] = 8.4, *p* < .006). There were no between or within-group changes for DBP. See Fig. 2 for a visual summary of significant results and Table 1 for detailed study results. 

The SFBT visit structure aligned with the 20-to-30-minute medical encounter (M = 24.69. SD = 3.98, Range = 15–30). There was minimal participant attrition with only 1 participant in the SFBT group dropping out of the study. In addition, patients in the SFBT intervention group had high acceptability (M = 4.92/5), stated the intervention was appropriate (M = 4.89/5), and indicated that the SFBT intervention was highly feasible (M = 4.91/5). Feasibility and acceptability measures are discussed further within our fidelity paper [25]

## Discussion

In this RCT of patients with depression and co-occurring chronic illness, participants who received a SFBT intervention, compared with TAU, achieved clinically meaningful improvements for depression (47% reduction, η2 = 0.35) and anxiety (43% reduction, η2 = 0.22) while increasing measures of well-being (24% increase, η2 = 0.15). In addition, there were trending effects for SBP and significant effects for a small sample for HbA1c. There were no significant effects of this short intervention on BMI or DBP. The SFBT intervention demonstrated efficacy with as little as three 15–30-minute sessions and therefore aligns with IC models to include the PCBH model. SFBT may be a sustainable, efficient, and cost-effective intervention for the management of depression and co-occurring chronic illness. These findings could have implications for clinics seeking to implement IC –perhaps via SFBT– within primary care settings.

Previous literature has examined CBT [36] and ACT [37, 38] for IC models within primary care settings, and other studies have examined SFBT interventions for addressing obesity and health behaviors within primary care [23]. An existing study found moderate effects for well-being/quality of life similar to the results from our study (η2 = 0.30 ) [37]. However, the effect sizes for depression (η2 = 0.08 ) and anxiety (η2 = 0.01 ) were small [37]. In comparison, our study produced moderate to high effect sizes for depression, anxiety, and well-being. Further, our study is the first to analyze the efficacy of SFBT interventions for treating depression and co-occurring chronic illness as a part of an IC model within a primary care setting. In addition, our study was the first to provide SFBT intervention at the dose and frequency that adheres to a warm handoff intervention (20–30 min encounter with 2–3 follow-up visits) [14]. This is significant as most fully IC models such as the primary care behavioral health (PCBH) model utilize warm handoff interventions. Our study was the first to examine depression and anxiety concurrently demonstrating that the SFBT intervention group had significantly decreased anxiety symptoms compared to the TAU group.

Our study is also the first to examine measures of flourishing. Human flourishing is a construct similar to well-being, and existing research has demonstrated its importance for population health, health promotion, and health prevention [39, 30]. There are, however, no identified studies that have analyzed the effects of a SFBT intervention within for an IC model and within a primary care context for the promotion of flourishing outcomes. Within our study, flourishing increased for those who engaged in the SFBT intervention. SFBT therefore shows promise to not only reduce depression and anxiety but to increase the experiences of human flourishing. This is an important finding for analyzing patient functionality and wellness in addition to symptom change.

An additional contribution is that our study was performed in an FQHC which often provides medical services to those who are of lower socioeconomic status and with more complex, comorbid disorders [40]. Our setting was, in addition, unique as it contains a rural population that typically does not have access to behavioral health interventions [41]. IC models attempt to expand the access of those who receive behavioral interventions by making care more accessible, and less stigmatizing. Our study demonstrates that SFBT interventions are effective at reducing psychological burdens but not metabolic illnesses for those who are from low-income and rural populations which has tremendous implications for expanding behavioral interventions for patients who do not regularly receive them.

### Limitations

There are limitations within this work that merit discussion. For example, the study was conducted over a short period (3 weeks), and there is therefore no way to determine whether treatment effects would persist after the SFBT intervention episode. Furthermore, the short time period may explain the lack of benefit in physical outcomes which usually take several weeks or months to manifest benefits. However, our study provides the context of treatment effects following an intervention created for an IC model (20–30 min; 1–3 sessions). Future research could replicate the existing study with more follow-up and increased assessment of survey data and biological measures. In addition, future research could utilize a Solomon design RCT (four groups) to better specify the efficient dosage (frequency and duration of intervention, utilization of booster sessions) needed for maximum therapeutic effect. Acquiring vital signs and HbA1c measures throughout the design was challenging with limited infrastructure (research staff, funds to perform laboratory testing) leading to a smaller sample for healthcare outcomes, **See** Table 1. Also, anxiety and depression are highly comorbid disorders, and this should be considered when interpreting the reductions in anxiety and depression. Regarding fidelity, our study did include self-report measures which could introduce bias. However, we triangulated data from chart reviews, clinician self-reports, and patient surveys to reduce bias and increase the rigor of our fidelity assessment.

## Conclusions

Among patients with co-occurring depression and chronic medical/psychiatric conditions in a FQHC in the Southeastern region of the United States, a SFBT intervention, compared with TAU, resulted in statistically significantly greater improvements in depression, anxiety, and overall well-being. In addition, our study demonstrates that a SFBT intervention dose of three 20–30 min weekly sessions is feasible to implement in primary care settings. Future research can incorporate a larger sample with more waves of data and longer follow up. In addition, performing the intervention within the context of a warm handoff may provide additional findings regarding the utility of SFBI within fully IC models.

### Electronic supplementary material

Below is the link to the electronic supplementary material.

Supplementary Material 1

Supplementary Material 2

Supplementary Material 3

## Data Availability

The datasets generated and/or analyzed during the current study are not publicly available due to privacy concerns but are available from the corresponding author on reasonable request.
