# Supplementary material for: Addressing depression and comorbid health conditions through solution-focused brief therapy in an integrated care setting: a randomized clinical trial
Source: BMC Prim Care. 2024 Aug 23;25:313. doi: 10.1186/s12875-024-02561-8 (PMC11342549; doi:10.1186/s12875-024-02561-8)
Supplement: Supplementary file 1 — Supplementary Material 1 [file 12875_2024_2561_MOESM1_ESM.docx]

Supplement 1: Intervention Workflow

Treatment as Usual (PCP, psychiatric medication)

**Perform all baseline measures. Remind of upcoming Perform all outcome measures.**

**Discuss treatment history. appointment. Discuss treatment plan.**

**Baseline Assessment Week 2 Check in Outcome Measures**

**Perform all baseline measures. SFBT #2 SFBT #3**

**SFBT Session #1 Schedule outcome visit. Perform all outcome measures.**

Treatment as Usual (PCP, psychiatric medication)
