# Supplementary material for: Addressing depression and comorbid health conditions through solution-focused brief therapy in an integrated care setting: a randomized clinical trial
Source: BMC Prim Care. 2024 Aug 23;25:313. doi: 10.1186/s12875-024-02561-8 (PMC11342549; doi:10.1186/s12875-024-02561-8)
Supplement: Supplementary file 2 — Supplementary Material 2 [file 12875_2024_2561_MOESM2_ESM.docx]

**Name and contact information**

Zach Cooper, LCSW, CADC-II, PhD Student

University of Georgia

School of Social Work

279 Williams Street

Athens, GA 30602

706-414-6278

zach.cooper@uga.edu

**Title**

Addressing Depression through Solution Focused Brief Therapy in Integrated Care Settings

**Description of proposed study**

Integrated behavioral healthcare (IBH) emerged to address the high prevalence of psychosocial issues endemic to primary care settings coupled with primary care provider’s discomfort in addressing psychosocial issues (Bikson et al., 2009; Loeb et al., 2012; Wise, 2012). IBH addresses the lack of psychosocial care in primary care settings by expanding the traditional healthcare team through the inclusion of a behavioral health provider (clinical social worker, licensed psychologist, etc.) (Robinson & Reiter, 2016). The behavioral health provider utilizes evidenced based interventions to support the healthcare team with addressing a wide range of healthcare concerns. Solution Focused Brief Therapy (SFBT) provides a promising treatment approach within IBH settings due to the high productivity standards within primary care and the efficient, solution based style foundational to SFBT (Blount & Bayona, 1994; Fraser et al., 2018; Strosahl, 1998). In addition, SFBT is strengths based and emphasizes patient centered approaches which primary care aspires to achieve. Despite the natural fit, there is a paucity of research regarding SFBT within integrated care settings in general, and for specific disease states. The purpose of this study is to assess the efficacy of SFBT within an IBH setting in the treatment of depression while assessing for commensurate improvement with traditional healthcare markers such as A1C, blood pressure, pulse, and weight (Elliott & Coventry, 2012). In addition, scaling questions will be utilized to assess for increase in core SFBT constructs to include self-awareness of strengths, future hope, and increased ability to problem solve. A pre-posttest experimental design will assess the differences between those receiving SFBT and treatment as usual across symptoms of depression, SFBT core attributes, and health outcomes.

**Statement of problem**

**The impact of depression**

Depressive disorders represent the most common disease states and produce significant influence for mortality and functional impairment (Gilman et al., 2017; Rovner et al., 1991). When depression is experienced with other diseases, there is a bidirectional impact on physical and mental health (Bush et al., 2001; Kanton et al., 2005; Meng et al., 2012; Roy & Lloyd, 2012). Depressive symptoms disrupt health and quality of life making it difficult to experience hope, purpose, and joy. Medical and mental health is connected is through the intersection of our daily life experiences and our psychology, which includes our thoughts, emotions, and biological processes (Kiecolt-Glaser et al., 2002; Vedhara et al., 1999). Life experiences influence cognitive and emotional states including symptoms of depression (Eaves & Rush, 1984; Rush et al., 1986) and neurobiology (Manji et al., 2001; Nemeroff, 2002; Nestler et al., 2002). These neurobiological processes facilitate changes in our neurochemistry influencing our immune and endocrine systems and impacting our health. Consequently, to effectively treat depression and its related consequences, healthcare systems must create systems that address depression wholistically and provide care and treatment across psychological, biological, and social systems.

**The disintegration of healthcare**

Despite the connection between psychosocial factors such as depression and general healthcare outcomes, the number of health centers providing whole person care is scant, and many individuals are not able to access behavioral health treatment. In fact, 57% of those with behavioral health disorders do not receive treatment, and 33% receive treatment only within primary care settings (Kathol, 2007). Primary care providers indicate that they are not comfortable with treating mental and behavioral health concerns (Bikson et al., 2009), and report limited time with patients due to high volumes within primary care settings (Jimenez et al., 2021; Loeb et al., 2012). Further complicating effective treatment in primary care stings, many patients do not follow through with referrals to specialty mental health and when they do, care is provided in a siloed and disintegrated fashion (Robinson & Reiter, 2016). IBH and SFBT both seek to address the problem of access to behavioral healthcare. IBH addresses the issue of access to behavioral healthcare through embedding a behavioral health provider into primary care teams and provide behavioral healthcare through a population health lens. SFBT addresses the problem of service access through a model of treatment that is more comprehensive and links patient needs across psychological, biological, and social systems to available resources using an empowering approach.

**Previous research in this area**

**Integrated care**

IBH first emerged in the 1970s with research beginning in the 1990s (Blount & Bayona, 1994). The majority of IBH models began within primary care settings to address the high prevalence of behavioral health needs seen in primary care settings (Hatala, 2012; Robinson & Reiter, 2016). To support the provision of behavioral health in primary care settings, IBH models emphasize a team approach incorporating a behavioral health professional with expertise in psychosocial care (clinical social worker, psychologist, etc.) within the traditional primary care team (Robinson & Reiter, 2016; Strosahl, 1998). Several treatment models have been studied within IBH contexts (Fraser et al., 2018). Existing research has reviewed the impact of IBH on functionality as well as with symptom presentation (Hunter et al., 2018). However, there is limited research using rigorous approaches including experimental designs to evaluate specific evidenced based treatments. While these rigorous approaches have examined approaches including Cognitive Behavioral Therapy (Cape et al., 2010) and Acceptance and Commitment Therapy (Glover et al., 2016; Kanzler et al., 2022), no studies to our knowledge examine and efficacy of SFBT within IBH settings using a rigorous experimental design.

**Solution focused therapy and integrated care settings**

Traditional primary care services focus on the assessment and treatment of biological symptoms. Consequently, primary care providers have challenge with caring for psychosocial needs and often indicate not having the time or expertise to address them (Loeb et al., 2012). In contrast, SFBT emphasizes patient strengths, focusing on what is going well with the individual and how they could mobilize their strengths to identify solutions to create a meaningful life. SFBT also emphasizes very important people in the patient’s life to increase social supports for the patient. The structure of SFBT sessions utilizes questions which prompt patient language that supports patients with identifying what is working in their lives and how to continue behavior associated with progress. Due to the emphasis on strengths and solutions, SFBT is much more efficient than problem-based therapy making it an ideal fit for the primary care setting. In addition, SFBT has significant research support (Gingerich et al., 2012; Gingerich & Peterson, 2013; Kim, 2008). SFBT also shows strong conceptual fit for IBH settings (Blount, 2019; González Suitt et al., 2016; Greenberg et al., 2001). Further research has examined outcomes from SFBT within medical settings including childhood obesity (McCallum et al., 2007) and positive health behaviors such as increased physical activity, sleep hygiene, and positive diet choices (Valve et al., 2013). While scant research suggests SFBT may be effective in primary care settings within specific domains, there remains a need for increased rigor of research designs examining SFBT outcomes in primary care settings and an expansion of known outcomes associated with SFBT delivery.

**SFBT, depression, and health outcomes**

SFBT has demonstrated efficacy for the treatment of depression, generally. Research from Gingerich and Eisengart (2000) reviewed multiple randomized control trials demonstrating a reasonable efficacy for SFBT in treating depression. However, to date we have identified no research that has assessed the efficacy of SFBT within IBH settings. This is a concerning gap in our knowledge, as SFBT has clear benefit for patient populations and a near-natural fit within IBH settings. In addition, there are a paucity of studies which examine the associations with additional health outcomes that are commonly influenced by depressive symptoms including hypertension, diabetes, and heart disease.

**Purpose of this study**

The purpose of this study is to examine the efficacy of SFBT in an integrated care setting for addressing depressive symptoms and health outcomes among patients with depression. The research questions are:

1. Is there a difference in core SFBT constructs (hope, connection with important people, strengths) between those who receive SFBT and those who receive treatment as usual?
2. Is there a change in symptoms of depression between those who receive SFBT and those who receive treatment as usual?
3. Is there a change in additional health-related outcomes associated with depression including between those who receive SFBT and those who receive treatment as usual?

**Method**

**Sample and procedure**

The proposed research will use a pretest-posttest experimental design where the intervention group will receive a standardized integrated care treatment protocol along with SFBT. The control group will only receive a standardized integrated care treatment protocol. Based on an existing partnership with the Principal Investigator, participants will be recruited from a primary care clinic in the state of Georgia, where the Principal Investigator has worked as a Manager of Behavioral Health for 4 years. Patients who have scored at or above 10 on the PHQ-9 will be considered as eligible to participate in the study. After intake and initial depression screening, any patients who have scored at or above 10 on the PHQ-9 will be considered as eligible to participate in the study. Following the consent process, individuals will be randomly assigned using a random number generator process to either the SFBT treatment condition or treatment as usual condition. Individuals in the SFBT condition will receive standard integrated care protocol, plus three sessions of SFBT. The control group will only receive the standard integrated care protocol from a primary care provider. A minimum of 50 participants will be recruited into this study. A power analysis using the software G*Power (Faul et al., 2009) shows that a sample of 48 participants will adequately power all statistical testing processes and permit the detection of medium to small effects (d = .20), given the design of the research. To ensure effective delivery of SFBT, the Principal Investigator and behavioral health provider will be enrolled in a Solution Focused Online Intensive Training program provided by The Institute for Solution Focused Therapy to gain a robust foundational understanding of SFBT and to ensure fidelity of the provision of SFBT within the research process (Vedder, 2022). The training is online, asynchronous and uses readings, lectures, and mock interviews with patients; participants are also tested on their knowledge via online test at the end of each module. In addition, a SFBT fidelity scale will also be used to ensure service delivery is consistent SFBT best practices (Lehmann & Patton, 2011). The scale will be utilized by the clinician as a self-assessment to ensure SFBT fidelity.

**Measures**

The dependent variables within the study will be 1) Symptoms of depression, 2) SFBT attributes, and 3) Health outcomes. Additional demographic measures will also be examined as potential control or moderating variables. The independent variable within the study will be the receipt of SFBT or treatment as usual (TAU).

*Symptoms of depression*. Depression will be measured at both pretest and posttest with the PHQ-9. The PHQ-9 is a standard assessment utilized in all federally qualified health centers and many primary care clinics and is therefore already embedded in the clinic workflow. The PHQ-9 has an established history of predictive validity and acceptable sensitivity (88%) and specificity (88%) (Kroenke et al, 2001).

*SFBT attributes.* These will be assessed at both pretest and posttest with scaled questions including hope, patient strengths, and connection to important people.

*Health outcomes.* These outcomes will be assessed through patient chart review at both pretest and posttest. These measures will include traditional healthcare markers such as blood pressure, weight, pulse, and A1C measurements (Elliott & Coventry, 2012).

*Flourishing.* Flourishing is a measure similar to well-being. The Flourishing index includes assesses several domains of flourishing including 1) life satisfaction, 2) mental and physical health, 3) meaning and purpose, 4) character and virtue, 5) close social relationships, and 6) financial and material stability.^47^ The Flourishing index has demonstrated sufficient reliability (α= .89) and predictive validity.^48^

*Demographic measures.* These measures will be collected only at baseline and will include age, gender, race, ethnicity, sexual orientation, education, income, and marital status.

**Data analysis plan**

To assess the utilization and efficacy of SFBT within an integrated care setting, patients who receive SFBT will be compared to those who receive TAU. Given the experimental nature of the research design, we will be using a factorial design ANOVA to examine changes within each group (SFBT vs. TAU) from pretest to posttest, and changes between groups (SFT vs. TAU) at pretest or posttest. Demographic measures will be examined to ensure adequate randomization between groups, as well as for their role in potential within-group effects associated with identified outcomes. All analyses will be completed using the statistical software packages of SPSS or STATA, which all members of the research team will be familiar with.

**Significance of study to SFBT field**

IBH seeks to address issues of access to behavioral healthcare through not only adding mental health providers to primary care teams but changing the workflows of mental health providers to match the population health approach seen in primary care settings (Blount & Bayona, 1994; Jimenez et al., 2021). The IBH approach dramatically expands the number of individuals who receive behavioral health services. In addition, the IBH team seeks to address the whole person focusing on strengths and solutions leading to more comprehensive and well-coordinated care. SFBT is an evidenced based treatment that fits within the primary care system. Assessing SFBT within IBH provides opportunity to drastically expand the utilization of SFBT and showcase the efficacy of SFBT for depression specifically, and for other health and mental health outcomes generally. Researching the impact of SFBT within IBH settings accomplishes a strategy to increase access to evidenced based intervention for large populations while assessing the efficacy of SFBT on a larger scale.

**Outline of how grant funds will be used**

**Proposed budget**

Visa Gift Cards ($20 for 50 Participants) $1,000

Training event venue for research clinician team $1,500

**Total $2,500**

**Budget justification**

*Visa Gift Cards ($20 for 100 Participants).* Expenses are required to compensate participants a reasonable about of money for their participation in the research. Gift card values will be reviewed and approved by the UGA IRB prior to beginning research.

*Training event venue for research clinician team.* To effectively train clinicians in SFBT processes, study recruitment and enrollment, the Principal Investigator will hold a day-long training event to provide instruction and oversight of clinical team processes as they related to the proposed research.

**Qualifications of the applicant**

The Principal Investigator is a PhD student who has been practicing as a mental health provider for 8 years, with 4 years of service in an integrated care setting. The Principal Investigator started and developed an integrated care clinic which he managed prior to enrolling as a PhD student. During that time, the Principal Investigator has gained significant experience practicing and teaching SFBT for depression and other health and mental health concerns. In addition, the Principal Investigator has published articles on integrated care models and has presented at national research conferences on the topic of integrated care. The Principal Investigator has completed multiple trainings through the Institute of Solution Focused Therapy and has access to several content experts for consultation.

**References**

Bikson, K., McGuire, J., Blue-Howells, J., & Seldin-Sommer, L. (2009). Psychosocial Problems in Primary Care: Patient and Provider Perceptions. *Social Work in Health Care*, *48*(8), 736–749. https://doi.org/10.1080/00981380902929057

Blount, A., & Bayona, J. (1994). Toward a system of integrated primary care. *Family Systems Medicine*, *12*(2), 171–182. https://doi.org/10.1037/h0089151

Bush, D. E., Ziegelstein, R. C., Tayback, M., Richter, D., Stevens, S., Zahalsky, H., & Fauerbach, J. A. (2001). Even minimal symptoms of depression increase mortality risk after acute myocardial infarction. *The American Journal of Cardiology*, *88*(4), 337–341. https://doi.org/10.1016/S0002-9149(01)01675-7

Cape, J., Whittington, C., Buszewicz, M., Wallace, P., & Underwood, L. (2010). Brief psychological therapies for anxiety and depression in primary care: Meta-analysis and meta-regression. *BMC Medicine*, *8*, 38. https://doi.org/10.1186/1741-7015-8-38

Eaves, G., & Rush, A. J. (1984). Cognitive patterns in symptomatic and remitted unipolar major depression. *Journal of Abnormal Psychology*, *93*(1), 31–40. https://doi.org/10.1037/0021-843X.93.1.31

Elliott, M., & Coventry, A. (2012). Critical care: The eight vital signs of patient monitoring. *British Journal of Nursing*, *21*(10), 621–625. https://doi.org/10.12968/bjon.2012.21.10.621

Fraser, M. W., Lombardi, B. M., Wu, S., de Saxe Zerden, L., Richman, E. L., & Fraher, E. P. (2018). Integrated Primary Care and Social Work: A Systematic Review. *Journal of the Society for Social Work and Research*, *9*(2), 175–215. https://doi.org/10.1086/697567

Gilman, S. E., Sucha, E., Kingsbury, M., Horton, N. J., Murphy, J. M., & Colman, I. (2017). Depression and mortality in a longitudinal study: 1952–2011. *CMAJ*, *189*(42), E1304–E1310. https://doi.org/10.1503/cmaj.170125

Gingerich, W. J., & Eisengart, S. (2000). Solution-Focused Brief Therapy: A Review of the Outcome Research*. *Family Process*, *39*(4), 477–498. https://doi.org/10.1111/j.1545-5300.2000.39408.x

Gingerich, W. J., Kim, J. S., Stams, G. J. J. M., & Macdonald, A. J. (2012). Solution-focused brief therapy outcome research. In *Solution-focused brief therapy: A handbook of evidence-based practice* (pp. 95–111). Oxford University Press.

Gingerich, W. J., & Peterson, L. T. (2013). Effectiveness of Solution-Focused Brief Therapy: A Systematic Qualitative Review of Controlled Outcome Studies. *Research on Social Work Practice*, *23*(3), 266–283. https://doi.org/10.1177/1049731512470859

Glover, N. G., Sylvers, P. D., Shearer, E. M., Kane, M.-C., Clasen, P. C., Epler, A. J., Plumb-Vilardaga, J. C., Bonow, J. T., & Jakupcak, M. (2016). The efficacy of Focused Acceptance and Commitment Therapy in VA primary care. *Psychological Services*, *13*(2), 156–161. https://doi.org/10.1037/ser0000062

González Suitt, K., Franklin, C., & Kim, J. (2016). Solution-Focused Brief Therapy With Latinos: A Systematic Review. *Journal of Ethnic & Cultural Diversity in Social Work*, *25*(1), 50–67. https://doi.org/10.1080/15313204.2015.1131651

Hatala, A. R. (2012). *The Status of the “Biopsychosocial” Model in Health Psychology: Towards an Integrated Approach and a Critique of Cultural Conceptions*. *2012*. https://doi.org/10.4236/ojmp.2012.14009

Hunter, C. L., Funderburk, J. S., Polaha, J., Bauman, D., Goodie, J. L., & Hunter, C. M. (2018). Primary Care Behavioral Health (PCBH) Model Research: Current State of the Science and a Call to Action. *Journal of Clinical Psychology in Medical Settings*, *25*(2), 127–156. https://doi.org/10.1007/s10880-017-9512-0

Jimenez, G., Matchar, D., Koh, G. C. H., Tyagi, S., Kleij, R. M. J. J. van der, Chavannes, N. H., & Car, J. (2021). Revisiting the four core functions (4Cs) of primary care: Operational definitions and complexities. *Primary Health Care Research & Development*, *22*. https://doi.org/10.1017/S1463423621000669

Kanton, W., Rutter, C., Simon, G., Lin, E., Ludman, E., Ciechanowski, P., Kinder, L., Young, B., & Korff, M. (2005). *The Association of Comorbid Depression With Mortality in Patients With Type 2 Diabetes | Diabetes Care | American Diabetes Association*. *28*(11), 2668–2672. https://doi.org/10.2337/diacare.28.11.2668

Kanzler, K. E., Robinson, P. J., McGeary, D. D., Mintz, J., Kilpela, L. S., Finley, E. P., McGeary, C., Lopez, E. J., Velligan, D., Munante, M., Tsevat, J., Houston, B., Mathias, C. W., Potter, J. S., & Pugh, J. (2022). Addressing chronic pain with Focused Acceptance and Commitment Therapy in integrated primary care: Findings from a mixed methods pilot randomized controlled trial. *BMC Primary Care*, *23*(1), 77. https://doi.org/10.1186/s12875-022-01690-2

Kiecolt-Glaser, J. K., McGuire, L., Robles, T. F., & Glaser, R. (2002). Emotions, Morbidity, and Mortality: New Perspectives from Psychoneuroimmunology. *Annual Review of Psychology*, *53*(1), 83–107. https://doi.org/10.1146/annurev.psych.53.100901.135217

Kim, J. S. (2008). Examining the Effectiveness of Solution-Focused Brief Therapy: A Meta-Analysis. *Research on Social Work Practice*, *18*(2), 107–116. https://doi.org/10.1177/1049731507307807

Lehmann, P., & Patton, J. D. (2011). The Development of a Solution-Focused Fidelity Instrument: A Pilot Study. In *Solution-Focused Brief Therapy*. Oxford University Press. https://doi.org/10.1093/acprof:oso/9780195385724.003.0019

Loeb, D. F., Bayliss, E. A., Binswanger, I. A., Candrian, C., & deGruy, F. V. (2012). Primary Care Physician Perceptions on Caring for Complex Patients with Medical and Mental Illness. *Journal of General Internal Medicine*, *27*(8), 945–952. https://doi.org/10.1007/s11606-012-2005-9

Manji, H. K., Drevets, W. C., & Charney, D. S. (2001). The cellular neurobiology of depression. *Nature Medicine*, *7*(5), 541–547. https://doi.org/10.1038/87865

McCallum, Z., Wake, M., Gerner, B., Baur, L. A., Gibbons, K., Gold, L., Gunn, J., Harris, C., Naughton, G., Riess, C., Sanci, L., Sheehan, J., Ukoumunne, O. C., & Waters, E. (2007). Outcome data from the LEAP (Live, Eat and Play) trial: A randomized controlled trial of a primary care intervention for childhood overweight/mild obesity. *International Journal of Obesity (2005)*, *31*(4), 630–636. https://doi.org/10.1038/sj.ijo.0803509

Meng, L., Chen, D., Yang, Y., Zheng, Y., & Hui, R. (2012). Depression increases the risk of hypertension incidence: A meta-analysis of prospective cohort studies. *Journal of Hypertension*, *30*(5), 842–851. https://doi.org/10.1097/HJH.0b013e32835080b7

Nemeroff, C. B. (2002). Recent advances in the neurobiology of depression. *Psychopharmacology Bulletin*, *36 Suppl 2*, 6–23.

Nestler, E. J., Barrot, M., DiLeone, R. J., Eisch, A. J., Gold, S. J., & Monteggia, L. M. (2002). Neurobiology of Depression. *Neuron*, *34*(1), 13–25. https://doi.org/10.1016/S0896-6273(02)00653-0

Robinson, P. J., & Reiter, J. T. (2016). *Behavioral Consultation and Primary Care*. Springer International Publishing. https://doi.org/10.1007/978-3-319-13954-8

Rovner, B., German, P., & Brant, L. (1991). *Depression and Mortality | JAMA | JAMA Network*. *265*(8), 993–996.

Roy, T., & Lloyd, C. E. (2012). Epidemiology of depression and diabetes: A systematic review. *Journal of Affective Disorders*, *142*, S8–S21. https://doi.org/10.1016/S0165-0327(12)70004-6

Rush, A. J., Weissenburger, J., & Eaves, G. (1986). Do thinking patterns predict depressive symptoms? *Cognitive Therapy and Research*, *10*(2), 225–235. https://doi.org/10.1007/BF01173727

Strosahl, K. (1998). Integrating behavioral health and primary care services: The primary mental health care model. In *Integrated primary care: The future of medical and mental health collaboration* (pp. 139–166). W. W. Norton & Company.

Valve, P., Lehtinen-Jacks, S., Eriksson, T., Lehtinen, M., Lindfors, P., Saha, M.-T., Rimpelä, A., & Anglé, S. (2013). LINDA – a solution-focused low-intensity intervention aimed at improving health behaviors of young females: A cluster-randomized controlled trial. *BMC Public Health*, *13*(1), 1044. https://doi.org/10.1186/1471-2458-13-1044

Vedder, J. (n.d.). *Foundations Solution-Focused · Solution-Focused Therapy Institute*. Retrieved July 11, 2022, from https://solutionfocused.net/foundations-course/

Vedhara, K., Fox, J. D., & Wang, E. C. (1999). The measurement of stress-related immune dysfunction in psychoneuroimmunology. *Neuroscience and Biobehavioral Reviews*, *23*(5), 699–715. https://doi.org/10.1016/s0149-7634(99)00012-3

Wise, M. J. (2012.). *The Prevalence of Psychosocial Issues in Primary Medical Care*. 70.
